# Supplementary material for: Quantification of stroke volume in a simulated healthy volunteer model of traumatic haemorrhage; a comparison of two non-invasive monitoring devices using error grid analysis alongside traditional measures of agreement
Source: PLoS One. 2021 Dec 23;16(12):e0261546. doi: 10.1371/journal.pone.0261546 (PMC8699736; doi:10.1371/journal.pone.0261546)
Supplement: S1 File — Background material and information sent to respondents in order to provide material to construct error grids. (DOCX) [file pone.0261546.s001.docx]

*
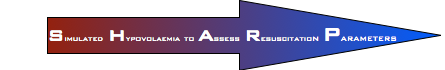
*

**Background**

We are asking for your help in a research study called SHARP (Simulated Hypovolaemia to Assess Resuscitation Parameters) which has finished data collection and is in the analysis phase. The study was funded by the Ministry of Defence, sponsored by King's College London and was carried out at the Clinical Research Facility in King's College Hospital. The aim of the study was to investigate the performance of various devices at detecting the presence of occult hypovolaemia in patients with simulated blood loss. Briefly we used a lower body negative pressure chamber technique to redistribute blood to the lower limb veins from the central venous circulation. We then compared the Stroke Volume (SV) measured at the Left Ventricular Outflow Tract by a fully accredited echocardiographer with that produced by other devices namely: NICOM bio reactance and USCOM supra sternal Doppler.

We have already conducted typical measures of correlation using linear regression and Bland - Altman analysis. Whilst these measures tell us about the mathematical degree of agreement they are less useful at telling us the clinical importance or relevance of the findings. To aid in this we are using a relatively new technique called Error Grid Analysis. This shows the degree of perceived clinical risk caused by disparity in the measured versus the actual results.

To conduct Error Grid Analysis requires the collection of a range of views from experienced specialist clinicians regarding the specific clinical scenario and the degree of acceptable risk. In this instance it involves answering two short questions after reading the scenario outlined below.

**Clinical Scenario**

Consider that you are managing a patient with traumatic injuries and a potential risk of hypovolemia.

- The patient is young and healthy with no obvious cardiovascular co-morbidity.
- You are using a stroke volume (SV) monitoring device to provide an indication of the onset of hypovolaemia and to guide resuscitation.
- The patient is initially euvolaemic but becomes hypovoalemic over time.
- For the purpose of this scenario assume that you are basing all haemodynamic management decisions solely on the change in stroke volume provided by the monitoring device.
- For the purposes of the scenario assume that the clinical intervention for a significant fall in SV will be the administration of blood products. Also assume that other potential falls in SV, such as that caused by an obstruction to cardiac output have been excluded.

**Question 1**

Consider a situation where the patient's stroke volume falls over time.

Please complete the table indicating what *percentage range* of fall in SV you would consider that:

- No action is currently required
- Action is indicated
- Action is essential

| **ACTION** | **% fall in SV** | **CODE** |
| --- | --- | --- |
| No action is currently required |  | A |
| Action is indicated |  | B |
| Action is essential |  | C |

**Question 2**

Now consider that the SV measurement is being made by a novel monitoring device and that the value produced by this monitor may differ from the actual SV, in other words there is a potential error in measurement.

**Please complete the table below categorizing the potential risk of patient harm resulting from any measurement error as: None / Mild / Moderate / Severe.**

These terms are necessarily subjective and will depend on you own views regarding the relative harm of omission or over administration of blood products in this clinical context.

The codes (A,B,C) from Question 1 indicate the actual values, which will vary between respondents. You can enter your values in the boxes if it aids completion but this is not essential.

| **Actual Stroke Volume reduction** | **Stroke Volume reduction indicated by monitor** | **Potential risk of patient harm resulting from measurement error** |
| --- | --- | --- |
| A | A | NONE |
|  | B |  |
|  | C |  |
| B | A |  |
|  | B | NONE |
|  | C |  |
| C | A |  |
|  | B |  |
|  | C | NONE |

Finally, please complete the following details. If you are an Intensivist please indicate whether you also undertake any current practice in another specialty.

**Speciality:**

**Years of Specialist Practice as a Consultant:**

Many thanks for your time

Sam Hutchings

Principal Investigator on behalf of the SHARP study investigators
