# Supplementary material for: Quantification of stroke volume in a simulated healthy volunteer model of traumatic haemorrhage; a comparison of two non-invasive monitoring devices using error grid analysis alongside traditional measures of agreement
Source: PLoS One. 2021 Dec 23;16(12):e0261546. doi: 10.1371/journal.pone.0261546 (PMC8699736; doi:10.1371/journal.pone.0261546)
Supplement: S2 File — Error grid questionnaire results from 15 respondents. Respondents first provided information on what fall in stroke volume from baseline would either require no action (Code A), possible action (Code B) or essential action (Code C)–these results are shown on the ACTUAL column of each table. Respondents were then asked to quantify the harm from a divergent measurement using the same range of stroke volumes–shown as DEVICE in the table. Harm was quantified numerically as None (0), Mild (2), Moderate (5) or Severe (10). Cumulative results are shown in a single table in which a range of actual versus measured falls in stroke volume are shown along with the perceived degree of harm from measurement error. The range of harm is from 0 (0 respondents thought that harm could occur) to 150 (all respondents thought that severe harm was likely). (DOCX) [file pone.0261546.s002.docx]

| **None** | **0** |
| --- | --- |
| **Mild** | **2** |
| **Moderate** | **5** |
| **Severe** | **10** |
| **RANGE** | **0-150** |

| **Respondent 1** | **DEVICE** | | |
| --- | --- | --- | --- |
| **ACTUAL** | **A** 0-12 | **B** 12-25 | **C** >25 |
| **A** 0-12 | **0** | **2** | **5** |
| **B** 12-25 | **2** | **0** | **5** |
| **C** >25 | **10** | **5** | **0** |

| **Respondent 2** | **DEVICE** | | |
| --- | --- | --- | --- |
| **ACTUAL** | **A** 0-5 | **B** 6-9 | **C** >10 |
| **A** 0-5 | **0** | **5** | **10** |
| **B** 6-9 | **5** | **0** | **5** |
| **C** >10 | **10** | **5** | **0** |

| **Respondent 3** | **DEVICE** | | |
| --- | --- | --- | --- |
| **ACTUAL** | **A** | **B** | **C** |
| **A** 0-30 | **0** | **10** | **10** |
| **B** 30-60 | **5** | **0** | **2** |
| **C** > 60 | **10** | **10** | **0** |

| **Respondent 4** | **DEVICE** | | |
| --- | --- | --- | --- |
| **ACTUAL** | **A** | **B** | **C** |
| **A 0-10** | **0** | **2** | **5** |
| **B 11-19** | **5** | **0** | **2** |
| **C > 20** | **10** | **5** | **0** |

| **Respondent 5** | **DEVICE** | | |
| --- | --- | --- | --- |
| **ACTUAL** | **A** | **B** | **C** |
| **A 0-10** | **0** | **2** | **5** |
| **B 10-20** | **2** | **0** | **2** |
| **C >20** | **5** | **2** | **0** |

| **Respondent 6** | **DEVICE** | | |
| --- | --- | --- | --- |
| **ACTUAL** | **A** | **B** | **C** |
| **A 0-5** | **0** | **2** | **5** |
| **B 6-14** | **5** | **0** | **5** |
| **C > 15** | **5** | **5** | **0** |

| **Respondent 7** | **DEVICE** | | |
| --- | --- | --- | --- |
| **ACTUAL** | **A** | **B** | **C** |
| **A 0-5** | **0** | **2** | **5** |
| **B 6-10** | **5** | **0** | **5** |
| **C > 11** | **10** | **10** | **0** |

| **Respondent 8** | **DEVICE** | | |
| --- | --- | --- | --- |
| **ACTUAL** | **A** | **B** | **C** |
| **A 0-10** | **0** | **5** | **10** |
| **B 10-24** | **5** | **0** | **10** |
| **C >25** | **10** | **10** | **0** |

| **Respondent 9** | **DEVICE** | | |
| --- | --- | --- | --- |
| **ACTUAL** | **A** | **B** | **C** |
| **A 0-20** | **0** | **5** | **10** |
| **B 20-49** | **2** | **0** | **5** |
| **C > 50** | **10** | **5** | **0** |

| **Respondent 10** | **DEVICE** | | |
| --- | --- | --- | --- |
| **ACTUAL** | **A** | **B** | **C** |
| **A 0-5** | **0** | **2** | **2** |
| **B 6-19** | **2** | **0** | **2** |
| **C > 20** | **5** | **5** | **0** |

| **Respondent 11** | **DEVICE** | | |
| --- | --- | --- | --- |
| **ACTUAL** | **A** | **B** | **C** |
| **A 0-10** | **0** | **2** | **2** |
| **B 11-19** | **5** | **0** | **2** |
| **C > 20** | **10** | **5** | **0** |

| **Respondent 12** | **DEVICE** | | |
| --- | --- | --- | --- |
| **ACTUAL** | **A** | **B** | **C** |
| **A 0-10** | **0** | **5** | **10** |
| **B 11-29** | **2** | **0** | **5** |
| **C >30** | **10** | **5** | **0** |

| **Respondent 13** | **DEVICE** | | |
| --- | --- | --- | --- |
| **ACTUAL** | **A** | **B** | **C** |
| **A 0-10** | **0** | **2** | **10** |
| **B 11-19** | **5** | **0** | **2** |
| **C >20** | **10** | **10** | **0** |

| **Respondent 14** | **DEVICE** | | |
| --- | --- | --- | --- |
| **ACTUAL** | **A** | **B** | **C** |
| **A 0-15** | **0** | **5** | **10** |
| **B 15-30** | **5** | **0** | **10** |
| **C> 30** | **10** | **5** | **0** |

| **Respondent 15** | **DEVICE** | | |
| --- | --- | --- | --- |
| **ACTUAL** | **A** | **B** | **C** |
| **A 0-10** | **0** | **2** | **2** |
| **B 11-29** | **2** | **0** | **5** |
| **C >30** | **10** | **2** | **0** |

| **MASTER CUMULATIVE**  **ABSOLUTE** | **DEVICE** | | | | | | | | | |
| --- | --- | --- | --- | --- | --- | --- | --- | --- | --- | --- |
| **ACTUAL** | **0-5** | **6-9** | **10-15** | **16-20** | **21-25** | **26-30** | **30-40** | **40-50** | **50-60** | **60-70** |
| **0-5** | **0** | **11** | **41** | **44** | **68** | **71** | **96** | **96** | **101** | **101** |
| **6-9** | **17** | **0** | **26** | **32** | **63** | **71** | **96** | **96** | **101** | **101** |
| **10-15** | **53** | **36** | **0** | **10** | **20** | **40** | **60** | **63** | **68** | **78** |
| **16-20** | **60** | **56** | **10** | **0** | **15** | **40** | **60** | **60** | **65** | **65** |
| **21-25** | **81** | **78** | **36** | **29** | **0** | **15** | **45** | **45** | **50** | **52** |
| **26-30** | **94** | **112** | **59** | **39** | **5** | **0** | **20** | **30** | **35** | **35** |
| **30-40** | **122** | **117** | **77** | **61** | **32** | **22** | **0** | **7** | **12** | **14** |
| **40-50** | **122** | **117** | **77** | **61** | **32** | **22** | **0** | **0** | **12** | **14** |
| **50-60** | **130** | **120** | **79** | **69** | **37** | **17** | **5** | **5** | **0** | **2** |
| **60-70** | **135** | **130** | **89** | **74** | **42** | **27** | **15** | **15** | **10** | **0** |
|  |  |  |  |  |  |  |  |  |  |  |

| **MASTER CUMULATIVE**  **PERCENTAGE** | **DEVICE** | | | | | | | | | |
| --- | --- | --- | --- | --- | --- | --- | --- | --- | --- | --- |
| **ACTUAL** | **0-5** | **6-9** | **10-15** | **16-20** | **21-25** | **26-30** | **30-40** | **40-50** | **50-60** | **60-70** |
| **0-5** | **0** | **7** | **27** | **29** | **45** | **47** | **64** | **64** | **67** | **67** |
| **6-9** | **11** | **0** | **17** | **21** | **42** | **47** | **64** | **64** | **67** | **67** |
| **10-15** | **35** | **24** | **0** | **6** | **13** | **27** | **40** | **42** | **45** | **52** |
| **16-20** | **40** | **37** | **6** | **0** | **10** | **26** | **40** | **40** | **43** | **43** |
| **21-25** | **54** | **52** | **24** | **19** | **0** | **10** | **30** | **30** | **33** | **35** |
| **26-30** | **62** | **75** | **39** | **26** | **3** | **0** | **13** | **20** | **23** | **23** |
| **30-40** | **81** | **78** | **51** | **40** | **21** | **15** | **0** | **5** | **8** | **9** |
| **40-50** | **81** | **78** | **51** | **41** | **21** | **15** | **0** | **0** | **8** | **9** |
| **50-60** | **87** | **80** | **53** | **46** | **25** | **11** | **3** | **3** | **0** | **1** |
| **60-70** | **135** | **130** | **89** | **74** | **42** | **27** | **15** | **15** | **10** | **0** |
|  |  |  |  |  |  |  |  |  |  |  |

**MASTER**

|  | **DEVICE** | | | | | |
| --- | --- | --- | --- | --- | --- | --- |
| **ACTUAL** | **0-5** | **6-9** | **10-14** | **15-19** | **20-29** | **>30** |
| **0-5** | **0** | **0** | **10** | **25** | **25** | **25** |
| **6-9** | **5** | **0** | **10** | **20** | **20** | **20** |
| **10-14** | **20** | **15** | **0** | **5** | **5** | **10** |
| **15-19** | **35** | **35** | **10** | **0** | **0** | **5** |
| **20-29** | **35** | **25** | **10** | **0** | **0** | **5** |
| **>30** | **40** | **30** | **20** | **5** | **5** | **0** |

**MIN 0**

**MAX 40**

**MASTER PERCENTAGE**

|  | **DEVICE** | | | | | |
| --- | --- | --- | --- | --- | --- | --- |
| **ACTUAL** | **0-5** | **6-9** | **10-14** | **15-19** | **20-29** | **>30** |
| **0-5** | **0** | **0** | **25** | **62.5** | **62.5** | **62.5** |
| **6-9** | **12.5** | **0** | **10** | **50** | **50** | **50** |
| **10-14** | **50** | **37.5** | **0** | **12.5** | **12.5** | **25** |
| **15-19** | **87.5** | **87.5** | **25** | **0** | **0** | **12.5** |
| **20-29** | **87.5** | **62.5** | **25** | **0** | **0** | **12.5** |
| **>30** | **100** | **75** | **50** | **12.5** | **12.5** | **0** |
