# Supplementary figures and images for: Quantification of stroke volume in a simulated healthy volunteer model of traumatic haemorrhage; a comparison of two non-invasive monitoring devices using error grid analysis alongside traditional measures of agreement
Source: PLoS One. 2021 Dec 23;16(12):e0261546. doi: 10.1371/journal.pone.0261546 (PMC8699736; doi:10.1371/journal.pone.0261546)

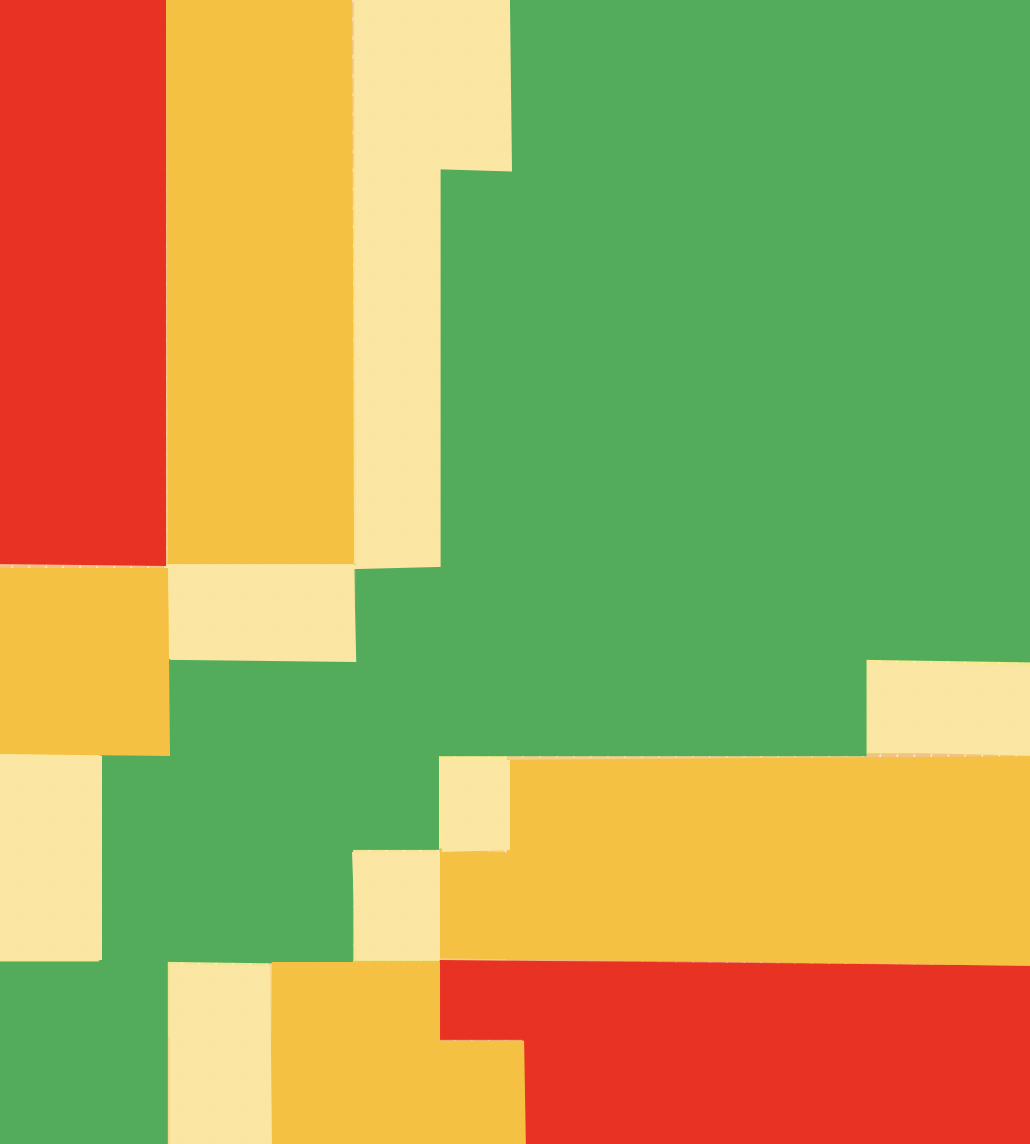

Supplement: S1 Fig — Smoothed polygon created from the Excel spreadsheet presented in File S3. Colours indicate degree of harm Red (Severe), Orange (Moderate), Yellow (Mild), Green (None). (PNG) [file pone.0261546.s004.png]
